# Effects of Adaptation on Discrimination of Whisker Deflection Velocity and Angular Direction in a Model of the Barrel Cortex

**DOI:** 10.3389/fncom.2018.00045

**Published:** 2018-06-12

**Authors:** Mainak J. Patel

**Affiliations:** Department of Mathematics, College of William and Mary, Williamsburg, VA, United States

**Keywords:** barrel cortex, whisker, deflection direction, deflection velocity, velocity discrimination, direction discrimination, feedforward inhibition

## Abstract

Two important stimulus features represented within the rodent barrel cortex are velocity and angular direction of whisker deflection. Each cortical barrel receives information from thalamocortical (TC) cells that relay information from a single whisker, and TC input is decoded by barrel regular-spiking (RS) cells through a feedforward inhibitory architecture (with inhibition delivered by cortical fast-spiking or FS cells). TC cells encode deflection velocity through population synchrony, while deflection direction is encoded through the distribution of spike counts across the TC population. Barrel RS cells encode both deflection direction and velocity with spike rate, and are divided into functional domains by direction preference. Following repetitive whisker stimulation, system adaptation causes a weakening of synaptic inputs to RS cells and diminishes RS cell spike responses, though evidence suggests that stimulus discrimination may improve following adaptation. In this work, I construct a model of the TC, FS, and RS cells comprising a single barrel system—the model incorporates realistic synaptic connectivity and dynamics and simulates both angular direction (through the spatial pattern of TC activation) and velocity (through synchrony of the TC population spikes) of a deflection of the primary whisker, and I use the model to examine direction and velocity selectivity of barrel RS cells before and after adaptation. I find that velocity and direction selectivity of individual RS cells (measured over multiple trials) sharpens following adaptation, but stimulus discrimination using a simple linear classifier by the RS population response during a single trial (a more biologically meaningful measure than single cell discrimination over multiple trials) exhibits strikingly different behavior—velocity discrimination is similar both before and after adaptation, while direction classification improves substantially following adaptation. This is the first model, to my knowledge, that simulates both whisker deflection velocity and angular direction and examines the ability of the RS population response to pinpoint both stimulus features within the context of adaptation.

## 1. Introduction

Synchronous spiking activity is a strategy commonly used by neuronal populations to encode and process information (Marthy and Fetz, 1992; Eckhorn, 1994; Gray, 1994; Laurent and Davidowitz, 1994; Friedrich et al., 2004; Patel et al., 2009, 2013; Patel and Joshi, 2015), and feedforward (or phase-delayed) inhibition is often employed to decode information encoded by population synchrony (Leitch et al., 1996; Deng and Rogers, 1998; Fricker and Miles, 2000; Pouille and Scanziani, 2001; Perez-Orive et al., 2002; Wehr and Zador, 2003; Benowitz and Karten, 2004; Blitz and Regehr, 2005; Mittmann et al., 2005; Jortner et al., 2007; Sridharan et al., 2011; Patel and Reed, 2013), due to its ability to robustly detect temporally coherent input (Bruno, 2011; Joshi and Patel, 2013; Patel and Joshi, 2013). The rodent barrel system prominently uses feedforward inhibition to process incoming information from the whiskers; each whisker transmits information to a whisker-specific population of ~250 thalamocortical (TC) cells (a barreloid), which excite a population <400 inhibitory fast-spiking (FS) cells and ~3,600 excitatory regular-spiking (RS) cells within the corresponding whisker-specific barrel. FS cells, in turn, supply potent, time-lagged inhibition to the RS cells, creating a narrow temporal window which constrains RS cell spike responses to TC input (Welker and Woolsey, 1974; Beaulieu, 1993; Kawaguchi and Kubota, 1993; Land et al., 1995; Keller and Carlson, 1999; Bruno and Simons, 2002; Sun et al., 2006; Cruikshank et al., 2007; Petersen, 2007; Bruno, 2011).

Whisker bending moment provides the major drive for primary mechanosensory cells (Peron et al., 2015; Campagner et al., 2016), and passive experiments employing whisker deflections in anesthetized animals show that two important features of primary whisker stimulation encoded within the corresponding barrel system are the velocity and angular direction of deflection (Bale and Maravall, 2018), which arises as a consequence of the encoding of bending moment (which is proportional to deflection angle) and the temporal derivative of the bending moment (which is proportional to deflection velocity) in barreloid afferents (Campagner et al., 2018). Thalamocortical cells encode a diverse array of stimulus features (Petersen et al., 2008), and a barreloid tends to encode deflection direction through the distribution of spiking activity across TC cells—barreloid TC cells are functionally divided into groups (with groups topographically arranged across the length of the barreloid) by direction preference (Timofeeva et al., 2003), and the magnitude of the spike response of a TC direction group diminishes as the angular direction of whisker deflection deviates from the preferred direction of the group toward the opposite direction 180° away (Pinto et al., 2000; Bruno and Simons, 2002; Temereanca and Simons, 2003). A barreloid encodes deflection velocity, on the other hand, through population synchrony; experiments show that different whisker deflection velocities lead to similar net spiking activity of the barreloid (as measured over the entire stimulus interval), while the spikes of TC cells within the barreloid become more temporally coherent with rising deflection velocity, leading to larger spike counts during the “ramp” phase of the stimulus (Pinto et al., 2000; Bruno and Sakmann, 2006; Temereanca et al., 2008). Thus, the net barreloid spike count is similar across deflection directions and velocities, with deflection direction encoded by the distribution of spikes across TC direction groups and deflection velocity encoded by the synchrony of TC spikes across the barreloid.

Within a barrel, FS cells tend to respond strongly to all whisker deflection velocities (Lee and Simons, 2004) and directions (Simons and Carvell, 1989; Bruno and Simons, 2002; Lee and Simons, 2004), and hence are neither velocity- nor direction-selective. Barrel RS cells, however, exhibit velocity and direction tuned responses; RS cells are clustered into domains by direction preference, with the arrangement of domains exhibiting a pinwheel structure (Bruno et al., 2003; Andermann and Moore, 2006; Kremer et al., 2011), and the spike response of an RS cell diminishes both as deflection velocity decreases and as deflection direction deviates from the preferred direction of the cell to the opposite direction 180° away (Pinto et al., 2000; Bruno and Simons, 2002; Lee and Simons, 2004; Wilent and Contreras, 2005). Interestingly, the direction tuning of an RS cell tends to sharpen with reductions in deflection velocity (Lee and Simons, 2004). Thus, the synchrony code employed by TC cells to represent deflection velocity is transformed into a rate code in RS cell responses (the temporal precision of RS cell spikes remains relatively constant across deflection velocities; Pinto et al., 2000; Bruno and Sakmann, 2006; Temereanca et al., 2008), while the rate code employed by TC cells to encode deflection direction is preserved in the activity of RS cells.

The dynamics of the barrel system adapt and change in an intriguing manner after low frequency (~20 Hz) repetitive whisker stimulation. If a whisker is deflected repeatedly with fixed velocity, the corresponding TC population response remains relatively unchanged, but synapses within the TC-FS-RS circuit weaken substantially, leading to a ~50% decrease in the potency of TC → RS excitation and a ~90% decrease in the magnitude of FS → RS inhibition. Furthermore, the spike response of an RS cell diminishes while the jitter in the timing of RS cell spikes rises (i.e., RS cell spikes display less temporal precision) following adaptation (Gabernet et al., 2005; Temereanca et al., 2008). Excitation and inhibition to an RS cell therefore both weaken following adaptation, but inhibition dampens substantially more than excitation. Interestingly, evidence suggests that the ability of the spike response of an RS cell to distinguish among deflection velocities may improve following adaptation (Wang et al., 2010; Adibi et al., 2013a,b; Liu et al., 2014).

In this study, I investigate a biophysical model of the TC, FS, and RS cells comprising a single whisker-specific barrel, with network connectivity and synaptic dynamics sharply constrained by experimental observations. This model was initially constructed to propose an explanation for experimental work showing that as the direction of whisker deflection deviates from the preferred direction of an RS cell, the peak amplitudes of net excitatory and net inhibitory input to the RS cell change minimally, while the timing of the peak in excitatory input shifts forward and approaches the (fixed) timing of the peak in inhibitory input, and hence the temporal window between the incoming net excitation and net inhibition diminishes for non-preferred deflection directions (Wilent and Contreras, 2005). The physiological mechanism underlying these intriguing observations has not been elucidated, and in prior work, I show that the model presented here can fully account for these empirical data via a simple and biologically plausible scheme. My prior investigation shows that, within the model, these observations arise naturally as a consequence of the presence of RS-RS synapses—since RS input to an RS cell is direction-independent and delayed relative to TC input, as TC input declines with non-preferred deflection directions, RS input “buffers” the decline in net excitation while causing the timing of the peak in excitation to shift from the TC input peak toward the (delayed) RS input peak (Patel, 2018).

In the current work, I use the model of Patel (2018) to study velocity and direction classification by not only the response of a single RS cell over multiple trials (a common experimental measure, since a fixed RS cell spikes only once or twice, or not at all, on a single trial), but also by the RS population response during the course of a single trial (a more biologically meaningful measure than single cell responses over multiple trials, though also more difficult to measure experimentally). Furthermore, since repetitive whisker deflection likely occurs frequently during natural roaming behavior, I study the effects of adaptation on the ability of RS cell responses to pinpoint stimulus features. Finally, since, unlike prior models of barrel cortex, the model presented here incorporates both stimulus features of deflection velocity and angular direction, I study how one stimulus feature affects discrimination of the other. This work provides explicit, experimentally testable hypotheses about direction and velocity discrimination, and the effects of system adaptation on identification of stimulus features, based on single-trial population responses, an important biological measure, since the animal must presumably make judgments based on overall barrel activity produced by individual whisker deflections rather than single-cell activity averaged over repeated trials of an identical stimulus.

## 2. Materials and methods

**Figure 1:**
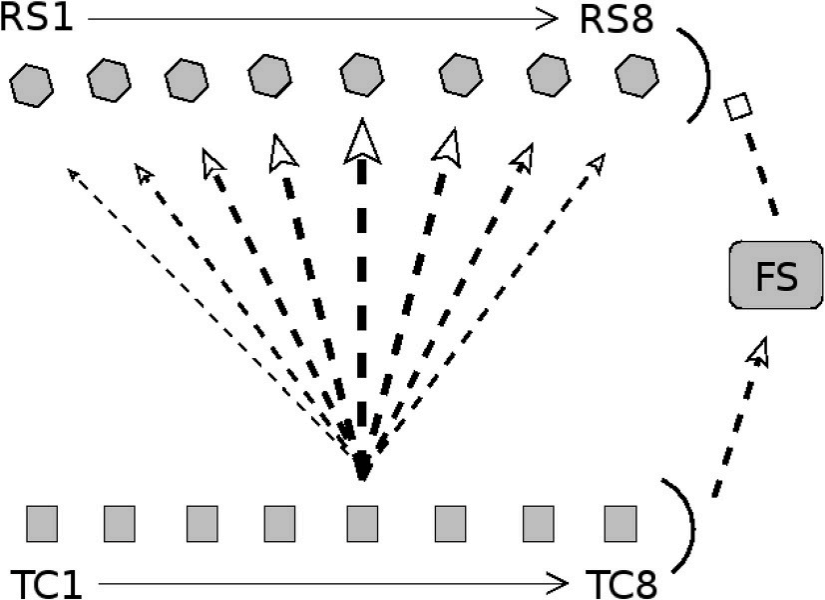
Diagram of connectivity in barrel model. Arrow heads indicate excitation; bar heads indicate inhibition. TC cells are divided into eight direction groups, with each group assigned a preferred angular direction of whisker deflection (1 = 180°, 2 = 225°, 3 = 270°, 4 = 315°, 5 = 0°, 6 = 45°, 7 = 90°, 8 = 135°). TC cells are not explicitly simulated; rather, spike times of TC cells are drawn from a distribution (stimulus velocity determines the synchrony of TC cell spikes, while stimulus direction determines the quantity of TC spikes across direction groups). RS cells are split into 8 direction domains, with each domain aligned to the TC direction group shown directly below. The density of TC → RS synapses depends on TC group-RS domain alignment; the diagram shows connection densities from the TC group with a direction preference of 0° (line/arrow thickness represents synapse density). Connectivity is analogous for other TC direction groups. TC cells uniformly excite a population of FS cells, which uniformly inhibit the RS cell population. Connectivity among RS cells is all-to-all. See text for details.

In this study, I construct a model of a single barreloid/barrel system of the rat barrel cortex corresponding to information arriving from a single whisker. The model consists of a thalamocortical (TC) cell population, a set of inhibitory fast-spiking (FS) cortical cells driven by the TC neurons, and a set of excitatory regular-spiking (RS) cortical cells that receive input from the TC and FS populations (see Figure 1). Connections among neurons are random but fixed, with direction- and cell type-specific connection probabilities.

### 2.1. Model connectivity

As observed experimentally (Land et al., 1995), the model barreloid consists of 240 TC cells, and since empirical observations show TC cell clustering by direction preference (Timofeeva et al., 2003), TC cells in the model are split into 8 direction groups of 30 cells each, with each group assigned a preferred whisker deflection direction (0°, 45°, 90°, 135°, 180°, 225°, 270°, 315°). The model consists of 100 FS cells and 160 RS cells (experimentally, a barrel is approximated to have <400 FS cells and ~3,600 RS cells; Welker and Woolsey, 1974; Beaulieu, 1993; Kawaguchi and Kubota, 1993; Keller and Carlson, 1999; Bruno and Simons, 2002). RS cells are split into 8 direction domains of 20 cells each (data suggest that RS direction domains are organized in a pinwheel structure; Andermann and Moore, 2006; Kremer et al., 2011), with each RS direction domain corresponding to a TC direction group.

Since FS cells have been shown to lack direction selectivity and respond strongly to all deflection directions (Simons and Carvell, 1989; Bruno and Simons, 2002; Lee and Simons, 2004), it is likely that TC input to FS cells is not direction selective (Swadlow and Gusev, 2002); hence, in the model I set a TC → FS connection probability of 0.65 for all TC direction groups (Bruno and Simons, 2002). The model has a FS → FS connection probability of 0.5 (FS → FS synapses in the model serve only to curtail the stimulus-induced FS population response) and a FS → RS connection probability of 1. An RS direction domain within a barrel has a horizontal span of ~100 μm (Keller and Carlson, 1999; Bruno and Simons, 2002; Bruno et al., 2003) (with individual RS cell dendritic arbors spanning ~200μm; Simons and Woolsey, 1984; Lübke et al., 2000), while a TC cell axon arborizes widely throughout the horizontal span of the full barrel (Jensen and Killackey, 1987), with the highest density of axon terminals within a ~200 μm horizontal range (Jensen and Killackey, 1987; Arnold et al., 2001); the extensive overlap of TC cell axon terminals with RS cell dendritic arbors suggests that a TC cell makes widespread synaptic connections to RS cells throughout a barrel, though synaptic densities vary with RS direction domain. Experimentally, the TC → RS connection probability has been estimated to be ~0.37 on average (with each RS cell receiving input from ~80 to 90 TC cells) (Bruno and Simons, 2002; Timofeeva et al., 2003; Bruno and Sakmann, 2006), and while RS cells are known to receive input from TC cells varying in direction preference (Timofeeva et al., 2003), experiments indicate that the likelihood of a TC → RS synapse varies considerably in a direction-dependent manner, with higher connection probabilities associated with greater alignment between TC and RS direction preferences (Bruno and Simons, 2002; Bruno et al., 2003; Furuta et al., 2011). Thus, in the model TC → RS synapses are direction specific, with the probability of a synapse varying with TC group-RS domain alignment. The connection probability for a TC direction group to an RS direction domain is set at 0.7, 0.5, 0.3, 0.15, 0.1 for TC group-RS domain alignments that differ by 0°, 45°, 90°, 135°, 180°, respectively (this leads to an average TC → RS connection probability of 0.35, with ~84 TC cells synapsing onto an RS cell). Though connectivity among RS cells within a barrel has not been well-characterized, evidence suggests that RS cells exhibit widespread arborization (Simons and Woolsey, 1984; Lübke et al., 2000) and that RS → RS synapses are likely to be abundant (Benshalom and White, 1986; Lübke et al., 2000)—hence, in the model I include all-to-all RS → RS connectivity.

### 2.2. Model equations

TC cells are not explicitly simulated (the times of stimulus-induced TC spikes are drawn from a distribution, as described below in the *Stimulus Simulation* section). The membrane potential of neuron *k, j* is governed by a reduced dimensional integrate-and-fire model of a cortical cell:

(1) $$\begin{array}{r} \frac{dV^{k,j}}{dt}=-g\left(V^{k,j}-V_{\text{rest}}\right)+I^{k,j}\left(t\right), \end{array}$$

where *k*∈ {fs,rs}, while *j*∈ {1,2,…,100} for *k* = fs and *j*∈ {1,2,…,160} for *k* = rs. *V*^*k, j*^ is the non-dimensional membrane potential, *g* = 0.05 ms^−1^ is the leak conductance, and *I*^*k, j*^(*t*) is the synaptic current (in ms^−1^). *V*_rest_ = 0 is the resting potential, and a spike is recorded when *V*^*k, j*^ → 1^−^, at which point *V*^*k, j*^ is instantaneously reset to *V*_rest_. A refractory period is simulated by holding *V*^*k, j*^ at *V*_rest_ for 2 ms following a spike. The integrate-and-fire equation has a membrane time constant of 20 ms, consistent with the experimentally observed ~17 ms time constant of RS cells (Gabernet et al., 2005). Details of the reduced dimensional model are given in (Tao et al., 2004).

A spike of a neuron presynaptic to neuron *k, j* leads to a jump in *I*^*k, j*^(*t*) followed by exponential decay, after a manually imposed synaptic latency. Let *n*^*k, j*^ denote the total number of presynaptic spikes that impinged upon neuron *k, j* during a trial. If the *r*^th^ presynaptic spike occurs at time $t_{r}^{k,j}$, and *m*∈ {tc,fs,rs} is the type of the presynaptic neuron, the current *I*^*k, j*^(*t*) induced in neuron *k, j* at time *t* is given by the following:

(2) $$\begin{array}{r} i_{r}^{k,j}\left(t\right)=\{\begin{array}{cc} 0\; & t<t_{r}^{k,j}+d_{m}^{k},\\ A_{m}^{k}e^{-\alpha_{m}^{k}\left(t-t_{r}^{k,j}-d_{m}^{k}\right)}\; & t\geq t_{r}^{k,j}+d_{m}^{k}. \end{array} \end{array}$$

(3) $$\begin{array}{r} I^{k,j}\left(t\right)=\overset{n^{k,j}}{\underset{r=1}{\sum }}i_{r}^{k,j}\left(t\right). \end{array}$$

$d_{m}^{k}$ denotes the synaptic delay, $\alpha_{m}^{k}$ dictates the decay rate, and $A_{m}^{k}$ indicates the amplitude of an input from a neuron of type *m*∈ {tc,fs,rs} to a neuron of type *k*∈ {fs,rs}. For the synaptic delay, $d_{\text{tc}}^{\text{fs}}=0$, $d_{\text{tc}}^{\text{rs}}=0$, $d_{\text{fs}}^{\text{fs}}=0$, $d_{\text{fs}}^{\text{rs}}=2$, $d_{\text{rs}}^{\text{rs}}=2$ ms. I introduced the delay parameter to match the experimental observation that a TC spike leads to an EPSP in the RS cell followed by an IPSP with a ~2 ms time lag (Gabernet et al., 2005), though the delay parameter does not qualitatively affect the dynamics of the model (due to model architecture, TC-induced FS spiking and TC-induced RS spiking must precede FS → RS and RS → RS input, respectively, ensuring that FS and RS input to an RS cell is delayed relative to TC input). For the decay rate, $\alpha_{\text{tc}}^{\text{fs}}=0.73$, $\alpha_{\text{tc}}^{\text{rs}}=0.75$, $\alpha_{\text{fs}}^{\text{fs}}=0.18$, $\alpha_{\text{fs}}^{\text{rs}}=0.18$, $\alpha_{\text{rs}}^{\text{rs}}=0.24$ ms^−1^. I chose these values to approximately match experimental data showing that TC synapses are fast and decay over a ~1-2 ms time scale, while FS synapses are slightly slower and decay over a ~5-6 ms time scale (Gabernet et al., 2005). The exact values of the decay rates (so long as synapses are fast, within the range of a few milliseconds) do not qualitatively affect model dynamics. For the amplitude, $A_{\text{tc}}^{\text{fs}}=0.3$, $A_{\text{tc}}^{\text{rs}}=0.06$, $A_{\text{fs}}^{\text{fs}}=0.1$, $A_{\text{fs}}^{\text{rs}}=0.04$, $A_{\text{rs}}^{\text{rs}}=0.008$ ms^−1^. I chose the amplitude parameters for approximate agreement with the following experimental observations on synaptic strengths within a barrel: (1) TC → RS synapses are relatively weak in comparison to potent TC → FS synapses (~30 incoming TC spikes are required to elicit a spike in an RS cell, while a few incoming TC spikes are capable of eliciting a spike in an FS cell) (Gabernet et al., 2005; Temereanca et al., 2008); (2) TC spikes elicit a ~4- to 8-fold larger EPSP in an FS cell than in an RS cell (Cruikshank et al., 2007); (3) the postsynaptic current in an RS cell induced by a whisker deflection is dominated by inhibition (in an RS cell, the ratio $\frac{\text{EPSC}}{\text{EPSC}+\text{IPSC}}=\sim 0.2$) (Gabernet et al., 2005). RS → RS synapses have not been characterized experimentally, and are assumed to be fast in the model.

Experimentally, adaptation after repeated whisker deflection leads to little change in the responses of TC cells, but leads to a net ~50% decrease in the amplitude of TC → RS excitation and a net 90% decrease in the magnitude of FS → RS inhibition (Gabernet et al., 2005; Temereanca et al., 2008). Thus, to simulate adaptation in the model, I multiply $A_{\text{tc}}^{\text{rs}}$ by 0.5 and $A_{\text{fs}}^{\text{rs}}$ by 0.1.

### 2.3. Stimulus modeling

Experimental data indicate that a whisker deflection tends to elicit at most one spike in a TC cell (Pinto et al., 2000); hence, in order to simulate a whisker deflection, I assign spike probabilities to TC cells (each TC cell spikes either 0 or 1 times) and I draw spike times for those TC cells that spike from an inverse Gaussian distribution (the inverse Gaussian distribution is the most concordant in shape with experimentally measured TC spike time distributions; Pinto et al., 2000).

Within a TC barreloid, experiments show that different whisker deflection velocities lead to similar net spike counts within the population; however, the synchrony of TC cell spikes varies directly with deflection velocity (Pinto et al., 2000; Bruno and Sakmann, 2006; Temereanca et al., 2008). To simulate the experimentally observed encoding of whisker deflection velocity by TC cell synchrony (rather than response magnitude), I simulate higher whisker deflection velocities by decreasing the standard deviation of the TC spike time distribution in the model, while leaving the spike probabilities for TC cells unchanged. This ensures that the net barreloid spike count in response to a whisker deflection does not depend on deflection velocity, but that barreloid spike counts are higher during the initial “ramp” phase of the deflection as velocity is increased (Pinto et al., 2000), due to a sharpening of the initial upward swing in the inverse Gaussian distribution as its standard deviation declines. The TC spike time distribution is set to have a mean of 10ms, and five velocities are simulated by setting the standard deviation of the TC spike time distribution (from high to low velocity) at 1, 1.25, 1.5, 1.75, or 2 ms. In manuscript figures, the inverse of the standard deviation of the TC spike time distribution is used as a stand-in for stimulus deflection velocity.

Experimental data further indicate that TC cells are clustered into groups by direction preference (Timofeeva et al., 2003), and that as the direction of a whisker deflection deviates away from the preferred direction of a TC direction group, the magnitude of the group's response diminishes, though the synchrony of the TC group's response does not change appreciably (Pinto et al., 2000; Temereanca and Simons, 2003). To simulate the encoding of whisker deflection direction by TC response magnitude, I set the TC spike probability within a given direction group at 0.8, 0.7, 0.4, 0.15, 0.1 to simulate a whisker deflection at a direction of 0°, 45°, 90°, 135°, 180°, respectively, away from the preferred direction of the TC direction group. This yields a tuning ratio (response to preferred direction/average response over all directions) of 1.86 for individual TC cells, concordant with experiment (Bruno and Simons, 2002). Thus, stimulus velocity in the model is encoded by the standard deviation of the TC spike time distribution, while stimulus direction is encoded by the distribution of spiking probabilities across TC direction groups.

### 2.4. Simulation and data analysis

All data presented in manuscript figures are averaged over 600 trials. Experiments indicate that the spike response of an RS cell to a preferred stimulus (if the cell responds at all) is low, with reported mean response values of 0.88 spikes/stimulus (Wilent and Contreras, 2005), 1.14 spikes/stimulus (Lee and Simons, 2004), 1.3 spikes/stimulus (Bruno and Simons, 2002), or 1–2 spikes/stimulus (Pinto et al., 2000). Such a low spike output implies that the response of a particular RS cell on a single trial can essentially be thought of as binary (spiking or no spiking), since spike quantity cannot encode for any stimulus feature. In the model, an RS cell tends to respond with 0 or 1 spikes to a stimulus (and occasionally with more than 1 spike). Due to the effectively binary nature of the single-trial response of an RS cell, in this study the response of an RS cell on a given trial is quantified in a binary fashion—either the RS cell does or does not spike on the trial. Hence, the probability that a RS cell spikes in response to a stimulus is calculated as the number of trials on which the RS cell spikes/total number of trials. Means and standard deviations are calculated using MATLAB software. Simulations are carried out using Euler's method with a time step of 0.01 ms.

## 3. Results

Figure 1 shows a schematic of model TC, FS, and RS cells comprising a single barreloid/barrel system. The model incorporates 240 TC cells divided into eight direction groups of 30 cells each (Land et al., 1995), with each direction group assigned a preferred direction of whisker deflection (Timofeeva et al., 2003). TC cells are not explicitly modeled; each cell spikes either 0 or 1 times per whisker deflection, and the times of TC spikes are drawn from a distribution similar to the experimentally observed TC spike time distribution (Pinto et al., 2000). To simulate a whisker deflection of a particular angular direction, TC cells within the corresponding direction group spike with high probability, while spike probability progressively diminishes in TC direction groups whose preferred directions deviate from the stimulus direction, with the lowest spike probability in the TC group with a preferred direction 180° away; the temporal distribution of TC cell spikes is fixed across direction groups (Pinto et al., 2000; Temereanca and Simons, 2003). To simulate different deflection velocities, the synchrony of TC cell spikes is varied (higher velocity corresponds to a smaller standard deviation in the distribution from which TC spike times are drawn), while the direction group-dependent TC spiking probabilities remain fixed (Pinto et al., 2000; Bruno and Sakmann, 2006; Temereanca et al., 2008). Thus, the net quantity of TC cell spikes (over all TC cells) does not vary with either stimulus velocity or direction—stimulus direction is represented by the distribution of spike counts across TC direction groups (with the TC spike time distribution fixed across direction groups), while stimulus velocity is represented by the standard deviation of the distribution of TC spike times. It is important to note that, in the model, while the net quantity of TC cell spikes over the entire duration of a trial does not vary with deflection velocity, increasing deflection velocity sharpens the initial “ramp” phase of TC cell spiking, substantially elevating barreloid spike counts immediately following stimulus onset (Pinto et al., 2000).

The 240 TC cells drive a small population of 100 FS cells (Simons and Carvell, 1989; Bruno and Simons, 2002; Swadlow and Gusev, 2002; Lee and Simons, 2004), and the FS cells inhibit a pool of 160 RS cells. The RS cells are organized into eight direction domains (Andermann and Moore, 2006; Kremer et al., 2011), with each RS domain aligned with a TC direction group (and assigned the corresponding direction label). The density of TC → RS synapses depends upon TC group-RS domain alignment – the probability that a TC cell synapses onto an RS cell diminishes as the direction domain of the RS cell deviates from the direction group of the TC cell, with the probability assuming a minimal value if the direction label of the RS domain differs by 180° from the direction preference of the TC cell (Bruno and Simons, 2002; Bruno et al., 2003; Furuta et al., 2011); the diagram in Figure 1 shows connection densities from the TC group with a preferred direction of 0° to RS direction domains (with analogous connectivity for other TC direction groups). RS → RS synapses within the model are all-to-all (Simons and Woolsey, 1984; Benshalom and White, 1986; Lübke et al., 2000); as described in prior work with this model (Patel, 2018) and elaborated upon within the Discussion section, RS → RS synapses within the model do not affect the spiking behavior of RS cells, and hence RS → RS synaptic transmission is not depicted in the results presented here. To simulate circuit adaptation in the model following repetitive low frequency (~20 Hz) whisker deflection, the strength of TC → RS synapses is decreased by 50% and the strength of FS → RS synapses is decreased by 90% (Gabernet et al., 2005; Temereanca et al., 2008). Model details and experimental justification of parameter values can be found in the Materials and Methods section.

### 3.1. Adaptation and velocity responses

**Figure 2:**
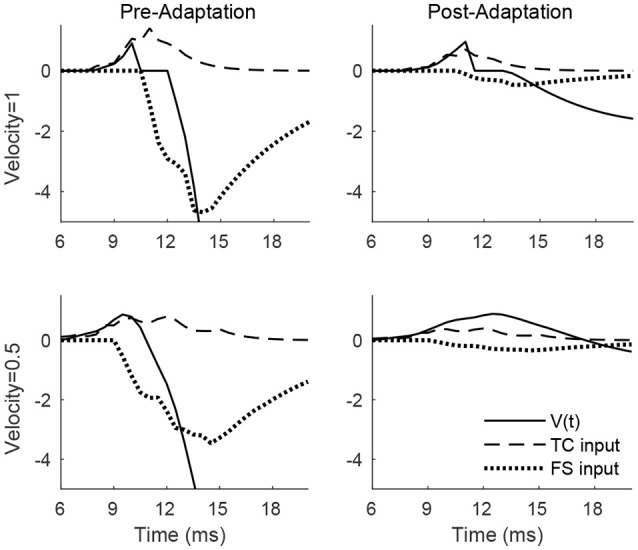
Membrane potential, net TC input current, and net FS input current to a sample RS cell within the 0° direction domain during a single trial. Data shown are for a high velocity (top row) and low velocity (bottom row) deflection, both before adaptation (left column) and after adaptation (right column). Stimulus deflection direction is fixed at 0° in all plots. The inverse of the standard deviation of the TC spike time distribution is used as a stand-in for deflection velocity.

Figure 2 shows the membrane potential, along with the TC and FS input currents, for a sample RS cell stimulated at its preferred deflection direction. For a high velocity deflection, TC input is synchronized and arrives within a narrow time window, with synchronized TC-induced inhibition from FS cells arriving a short time later; prior to adaptation, TC input is strong but dwarfed in peak magnitude by FS inhibition, ensuring that an RS spike response can only occur within a short temporal window prior to the arrival of inhibition, while following adaptation, TC input is moderately weaker but FS input is weakened far more dramatically, and hence TC input is less efficacious at driving the RS cell but is capable of impacting RS cell activity over a longer time window (since FS inhibition is too weak to sharply curtail RS cell activity upon its arrival). Prior to adaptation, the ratio $\frac{\text{EPSC}}{\text{EPSC+IPSC}}$ of the peak magnitudes of excitation and inhibition is 0.23, while following adaptation the ratio rises to 0.6, consistent with experimental observations (Gabernet et al., 2005). For a low velocity deflection, the net (time-integrated) TC input to the RS cell is unchanged but is less synchronized and spans a broader temporal window (which makes it less effective at eliciting an RS cell spike), with FS inhibition arriving shortly after TC excitation; prior to adaptation, the potent FS inhibition (triggered shortly after the inception of TC activity due to strong TC → FS synapses; Gabernet et al., 2005) sharply curtails excitation and constrains the RS cell to fire within a small time window, while after adaptation the drastically weakened inhibition is unable to effectively oppose the moderately weakened excitation, resulting in a more prolonged time window within which TC excitation can influence the RS cell. Consistent with experiment (Gabernet et al., 2005), the ratio $\frac{\text{EPSC}}{\text{EPSC+IPSC}}$ prior to and following adaptation changes from 0.20 to 0.56.

**Figure 3:**
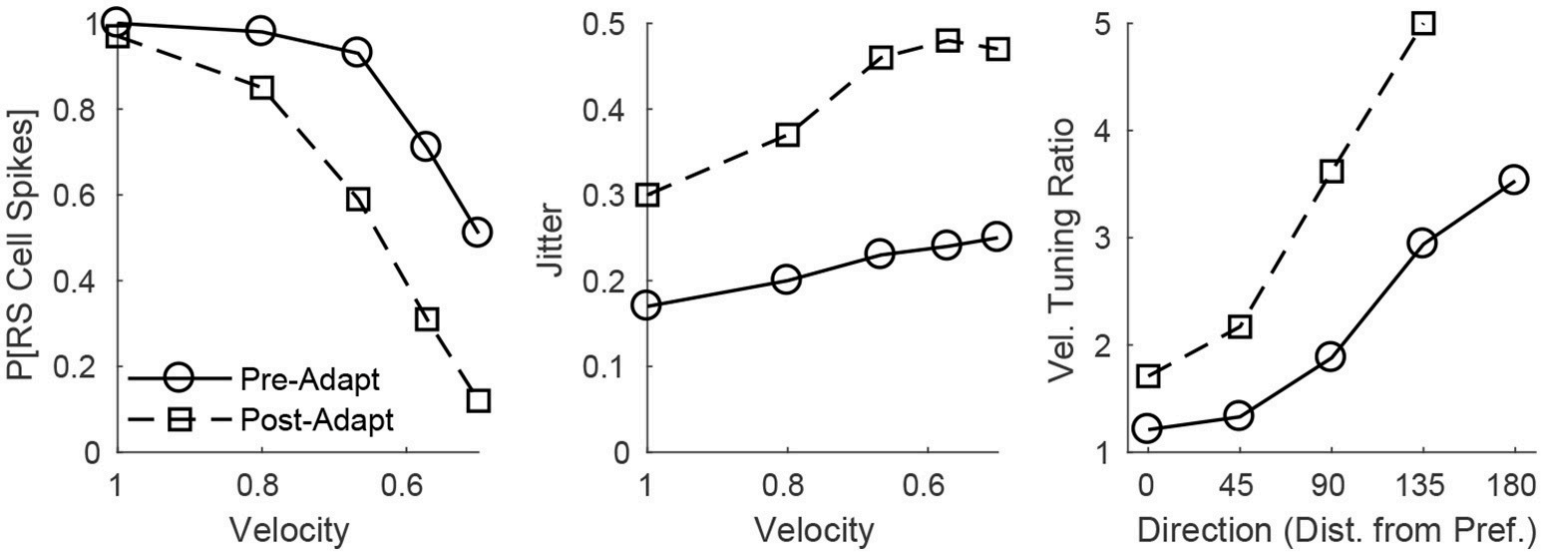
Behavior of an RS cell within the 0° direction domain to deflections of varying velocity, before and after adaptation. The left panel shows the probability that the RS cell spikes, while the middle panel shows the jitter (standard deviation) in the timing of the RS cell spike, as a function of deflection velocity (deflection direction is fixed at 0°). The right panel shows the velocity tuning ratio (response to highest velocity/average response over all velocities) of the RS cell as a function of deflection direction (data are averaged over deflection directions equidistant from preferred). Data are gathered over 600 trials.

The adaptation-induced changes in RS cell input dynamics discussed above have consequences in terms of the velocity dependence of an RS cell's spike response (Figure 3). Figure 3 (left) shows that, both pre- and post-adaptation, the spike response of an RS cell exhibits velocity tuning (since lowering TC synchrony results in a less effective excitatory drive to RS cells); following adaptation, however, the spike response diminishes at any fixed velocity (due to a reduction in the magnitude of the excitatory drive from TC cells), though the steeper decline in the spike response of the RS cell with decreasing velocity post-adaptation vs. pre-adaptation suggests that velocity tuning may sharpen following adaptation. This is likely a consequence of the non-linearity imposed by the spike threshold – adaptation leads to a fixed percentage reduction in the peak amplitude of excitatory input to an RS cell, and so the decline in TC synchrony with decreasing velocity has a greater impact on the probability of an RS cell spike after adaptation than before adaptation. Figure 3 (right) depicts the velocity tuning ratio (response to highest velocity/average response to all velocities) of the RS cell as a function of deflection direction, showing that velocity selectivity increases after adaptation (for any deflection direction). Additionally, Figure 3 (right) shows that velocity selectivity increases as deflection direction deviates from the preferred direction of the RS cell to the opposite direction 180° away; this is again due to the threshold non-linearity – as deflection direction deviates away from the preferred direction of the RS cell, the number of TC spikes impinging upon the RS cell diminishes, leading to a reduction in the peak amplitude of net excitation received by the RS cell (and hence the decrease in TC synchrony with reductions in velocity have a greater impact on the probability that the RS cell spikes).

Figure 3 (middle) shows that, in accordance with experiment (Gabernet et al., 2005; Temereanca et al., 2008), the jitter in the timing of a stimulus-induced RS cell spike changes little with deflection velocity but increases after adaptation. Prior to adaptation, powerful time-lagged inhibition from RS cells creates a narrow temporal window within which TC excitation can elicit an RS cell spike – decreasing deflection velocity alters the synchrony of TC input, hence reducing the probability that the RS cell will spike, but if the RS cell emits a spike, the spike is constrained to occur within this narrow time window (leading to a relatively stable jitter in spike timing). Following adaptation, the drastic dampening of inhibition from FS cells prolongs the time window over which TC input can trigger an RS cell spike (leading to a larger jitter in spike timing), and while decreasing deflection velocity broadens the temporal span of net TC input, the bulk of TC input tends to occur early after the stimulus (leading to only a modest increase in jitter with decreases in deflection velocity).

### 3.2. Adaptation and direction responses

**Figure 4:**
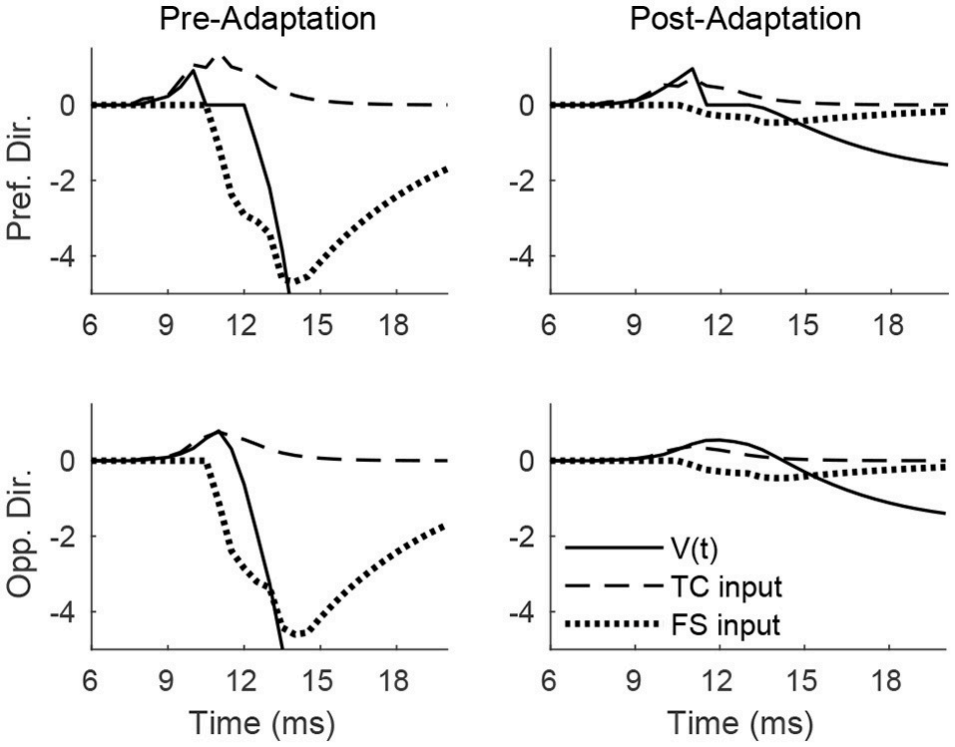
Membrane potential, net TC input current, and net FS input current to a sample RS cell within the 0° direction domain during a single trial. Data shown are for a deflection at the preferred (top row) and opposite-to-preferred (bottom row) deflection direction, both before adaptation (left column) and after adaptation (right column). Stimulus deflection velocity is fixed at 1 in all plots (the inverse of the standard deviation of the TC spike time distribution is used as a stand-in for deflection velocity).

Figure 4 shows the pre- and post-adaptation membrane potential, net TC input current, and net FS input current for a sample RS cell within the 0° direction domain, for a stimulus at the preferred and opposite-to-preferred deflection direction of the cell. Since deflection velocity is fixed, the temporal synchrony of TC input remains the same in all cases. For a deflection at the preferred direction of the cell, prior to adaptation the amplitude of TC input is large, leading to a high probability of an RS cell spike within the narrow time window prior to the arrival of potent FS inhibition, while after adaptation the probability of an RS spike is diminished, since TC input is diminished in magnitude (though TC excitation is capable of exerting influence over the RS cell over a longer time span, due to the drastic reduction in the potency of FS inhibition). For a deflection at the opposite direction, prior to adaptation the reduction in the peak amplitude of TC input yields a relatively low probability of an RS cell spike (with the spike constrained to occur within the window of unopposed excitation), while after adaptation the further reduction in TC input magnitude results in a further diminishing of the probability of an RS cell spike (though the inefficacious inhibition allows a broader time window for the occurrence of a spike).

**Figure 5:**
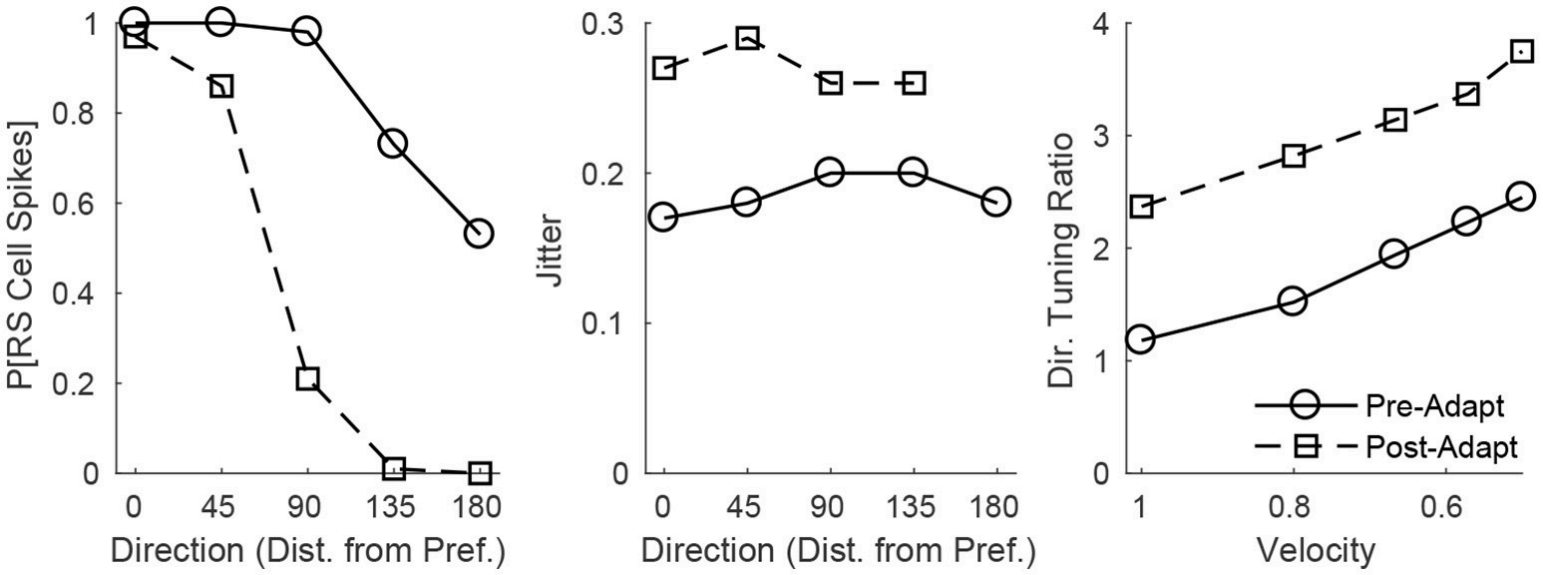
Behavior of an RS cell within the 0° direction domain to deflections of varying angular direction, before and after adaptation. The left panel shows the probability that the RS cell spikes, while the middle panel shows the jitter (standard deviation) in the timing of the RS cell spike, as a function of deflection direction (data are averaged over deflection directions equidistant from preferred; deflection velocity is fixed at 1). The right panel shows the direction tuning ratio (response to preferred direction/average response over all directions) of the RS cell as a function of deflection velocity. The inverse of the standard deviation of the TC spike time distribution is used as a stand-in for deflection velocity. Data are gathered over 600 trials.

These dynamics are manifested in the direction dependence of an RS cell's response (Figure 5). Figure 5 (left) shows that as deflection direction deviates from preferred, the probability of an RS cell spike declines, with a dramatically steeper decline following adaptation (since altering deflection direction away from preferred and adaptation both reduce the peak amplitude of TC input, this results in a compound effect that, combined with the effect of the non-linearity imposed by the spike threshold, yields a steeper decline in the probability of an RS cell spike with non-preferred stimuli after adaptation in the case of direction stimuli than in the case of velocity stimuli). The steeper decline with non-preferred stimuli following adaptation suggests that the response of the RS cell may exhibit sharper direction tuning post-adaptation vs. pre-adaptation; this is borne out in Figure 5 (right), which shows the direction tuning ratio (response to preferred direction/average response over all directions) of the RS cell as a function of deflection velocity. Additionally, and in accordance with experimental measurements of direction tuning ratios (Bruno and Simons, 2002; Lee and Simons, 2004; Wilent and Contreras, 2005), the direction tuning ratio of an RS cell in the model rises with decreasing deflection velocity (this is again due to the spike threshold non-linearity – the drop in TC synchrony with decreasing velocity leads to a smaller likelihood that the RS cell reaches threshold, and hence the decline in peak amplitude of TC input for non-preferred deflection directions has a greater impact on the probability of an RS cell spike for lower deflection velocities). Furthermore, Figure 5 (middle) shows that the jitter in the timing of the RS cell spike is relatively stable across deflection directions but increases following adaptation – prior to adaptation, potent FS inhibition constrains an RS cell spike to occur within a narrow temporal window, while after adaptation inhibition is drastically weakened and TC input can elicit a spike within a broader time span.

### 3.3. Velocity and direction discrimination

The results above on velocity and direction tuning ratios indicate that the spike response of an RS cell is better able to discriminate among deflection velocities and directions post-adaptation as opposed to pre-adaptation, and that velocity discrimination improves for non-preferred deflection directions while direction discrimination improves for lower deflection velocities. However, RS cells tend to spike at most once per stimulus, both in the model studied here and in empirical observations (Pinto et al., 2000; Bruno and Simons, 2002; Wilent and Contreras, 2005), and hence the velocity and direction tuning results presented above are based upon the *probability* that an RS cell spikes in response to a particular stimulus, a quantity that is computed through repeated stimulus presentation over multiple trials. An animal, on the other hand, must pinpoint deflection direction and velocity based upon a single trial, and the fact that an RS cell spikes ~0–2 times per stimulus implies that the spike response of a single RS cell is of little utility in a biological setting in direction or velocity discrimination. In a natural (single-trial) setting, an animal must therefore rely upon the spiking activity of a population of RS cells to extract information about stimulus direction or velocity, an issue that has received little experimental attention, though the utility of RS population responses is suggested by experimental work showing that pairs of RS cells convey more information about angular direction than single cells (Bale and Petersen, 2009).

**Figure 6:**
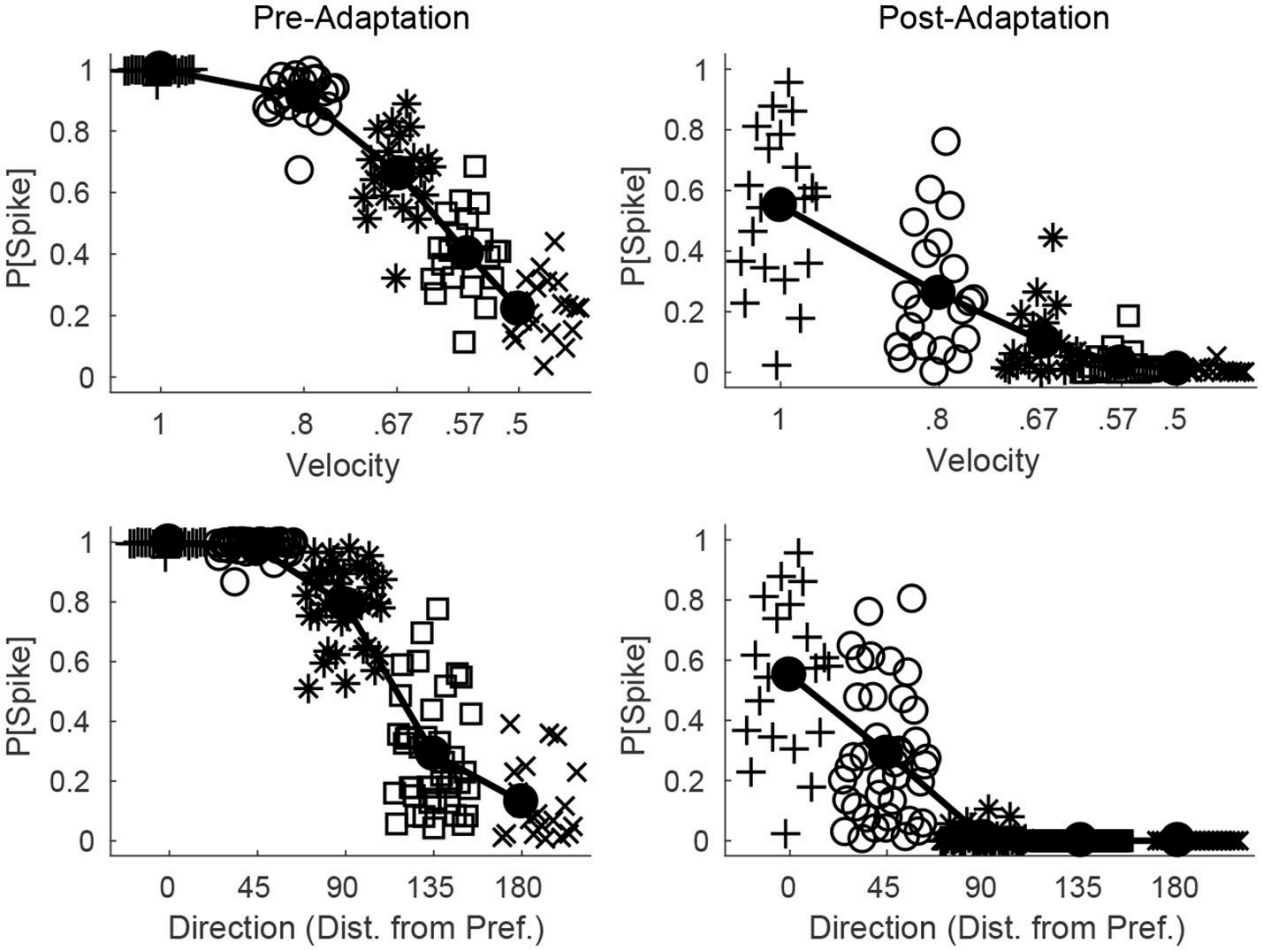
Direction and velocity dependence of RS population responses, before and after adaptation. The top row shows the probability that an RS cell spikes for RS cells within one direction domain as a function of deflection velocity, before (left) and after (right) adaptation (deflection direction is fixed at the preferred direction of the domain). The bottom row shows the probability that an RS cell spikes for RS cells within one direction domain as a function of deflection direction, before (left) and after (right) adaptation (data from directions equidistant from the preferred direction of the domain are aggregated; deflection velocity is fixed at 1). For a particular symbol type, data points represent the responses of individual RS cells within the domain, and symbol type is varied with velocity of the simulated deflection (hence there are 20 data points for each velocity; top row) or direction of the simulated deflection (since data are aggregated over directions equidistant from preferred, there are 40 data points for the 45°, 90°, 135° cases and 20 data points for the 0°, 180° cases; bottom row). The solid line shows the mean response of the RS direction domain. Data are gathered over 600 trials.

Figure 6 shows the probability of a spike for all RS cells within a fixed direction domain as a function deflection velocity (top row) or direction (bottom row), before (left column) and after (right column) adaptation. Prior to adaptation, high velocity or preferred direction stimuli lead to relatively homogeneous spiking behavior in the RS direction domain (all cells within the domain tend to spike with high probability), while as deflection velocity is lowered or deflection direction deviates from preferred the mean response of the domain diminishes but there is greater variability in the behavior of individual RS cells within the domain. Following adaptation, the mean response of the domain diminishes for all stimuli; however, for high velocity or preferred direction stimuli there is substantial variation in the responses of individual RS cells within the domain, while as deflection velocity is lowered or deflection direction deviates from preferred the variability in the responses of individual RS cells within the domain diminishes (for low velocity or non-preferred direction stimuli RS cells within the domain tend to spike with very low or zero probability). Thus, while adaptation increases response variability for high velocity or preferred direction stimuli, it precludes responses (and hence reduces response variability) for low velocity or non-preferred direction stimuli, which may have complex consequences for single-trial velocity or direction discrimination based upon responses of a population of RS cells.

**Figure 7:**
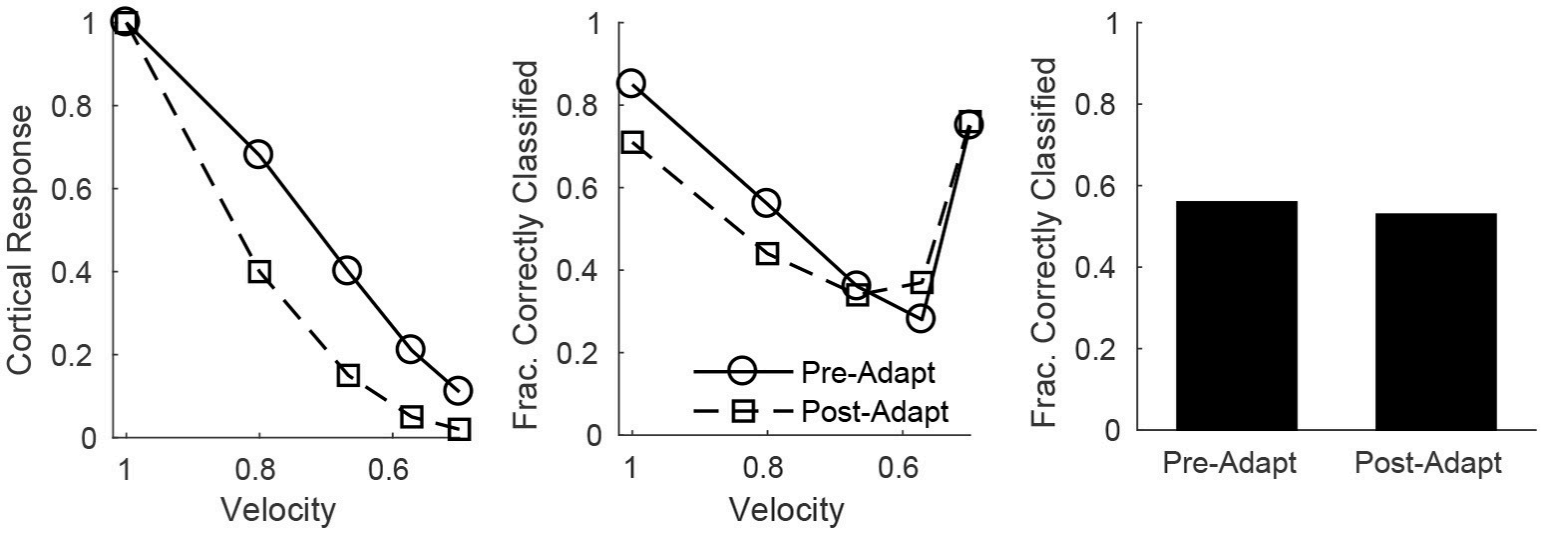
Velocity discrimination by the net cortical RS response, before and after adaptation. **Left**: Mean net cortical response as a function of deflection velocity, normalized by the response at a velocity of 1 (deflection direction is fixed). Net cortical response is defined as the total number of RS cell spikes in a trial; the mean is calculated over 600 trials. **Middle**: Fraction of correctly classified trials of each velocity (600 trials per velocity). The midpoints between the mean net cortical responses for adjacent velocities are set as classification cutoffs; a trial is defined as correctly classified if the net cortical response falls between the upper and lower cutoffs. **Right**: Data on fraction of correctly classified trials aggregated over all velocities (3,000 total trials; 600 trials of each velocity). The inverse of the standard deviation of the TC spike time distribution is used as a stand-in for deflection velocity.

Since all RS cells exhibit the same velocity dependence (spike probability diminishes with decreasing velocity), it is reasonable to assess population velocity discrimination using the aggregate response of all cortical RS cells. Figure 7 (left) shows the trial-averaged net response of all cortical RS cells as a function of stimulus deflection velocity (deflection direction is fixed, though irrelevant since the net response sums over all RS direction domains) – the mean net response diminishes substantially with decreases in deflection velocity, with smaller responses following adaptation. While this is suggestive of the ability of the cortical RS population to discriminate among deflection velocities (both pre- and post-adaptation), this is a trial-averaged measure—a more biologically meaningful measure is the ability of the cortical RS population to classify stimulus velocity on a single-trial basis. Figure 7 (middle) shows, using a linear classifying scheme, the fraction of correctly classified trials for stimuli of various velocities; interestingly, single-trial velocity classification by the RS population exhibits little change following adaptation (though classification is slightly more accurate for high velocity stimuli before adaptation and for low velocity stimuli following adaptation). This is a consequence of the balance between response variability among RS cells and the difference in the average population response for different deflection velocities, as suggested by Figure 6 (top row). For high velocities, the pre-adaptation difference in mean population response for different velocities is low but is counteracted by low variability in the responses of individual RS cells (allowing a relatively high classification rate), while the post-adaptation difference in mean population response for different velocities is high but this is effectively negated by the corresponding high variability in the responses of individual RS cells (leading to a similar classification rate). For low velocities, the high pre-adaptation difference in mean population response for different velocities is accompanied by high variability in the responses of individual RS cells, while the post-adaptation difference in mean population response for different velocities is low but there is also low variability in the responses of individual RS cells (leading to similar classification rates in the two scenarios). Thus, despite the earlier results showing that single-cell velocity tuning sharpens following adaptation, population data show that, due to the interaction between response variability among individual RS cells and the velocity dependence of the mean population response, single-trial velocity classification by the cortical RS population actually changes little with adaptation (Figure 7, right).

**Figure 8:**
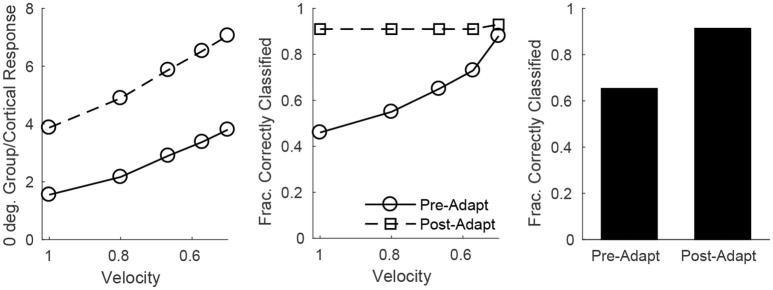
Direction discrimination by the cortical RS response, before and after adaptation, for a stimulus deflection direction of 0°. **Left**: Mean response of the 0° RS direction domain divided by the mean cortical response as a function of deflection velocity. Mean response is defined as number of spikes per cell averaged over RS cells (either cells in the 0° direction group or the entire cortex) in a trial; data shown are averaged over 600 trials. **Middle**: Fraction of correctly classified trials as a function of deflection velocity (600 trials per velocity). The midpoint between the mean response of the 0° RS direction domain divided by the mean cortical response (averaged over 600 trials) and the mean response of the 45°/315° RS direction domain divided by the mean cortical response (averaged over 600 trials) is set as the classification cutoff; a trial is defined as correctly classified if the mean response of the 0° RS direction domain divided by the mean cortical response for the trial exceeds the cutoff. **Right**: Data on fraction of correctly classified trials aggregated over all velocities (3,000 total trials; 600 trials of each velocity). The inverse of the standard deviation of the TC spike time distribution is used as a stand-in for deflection velocity.

On the other hand, single-trial direction discrimination by the cortical RS population exhibits strikingly different behavior before and after adaptation. Since RS cells are functionally and anatomically divided into domains by direction selectivity (Pinto et al., 2000; Bruno and Simons, 2002; Bruno et al., 2003; Lee and Simons, 2004; Wilent and Contreras, 2005), it is reasonable to assume that, biologically, the response of the RS direction domain whose preferred direction aligns with the deflection direction of the stimulus, in comparison to the responses of other direction domains, is the primary variable used by the barrel cortex to pinpoint the angular direction of whisker deflection. In order to assess direction discrimination in the model, I therefore fix stimulus deflection direction at 0° and study the response of the 0° RS direction domain relative to the net cortical response. Figure 8 (left) shows the (trial-averaged) response of the 0° direction domain divided by the net cortical response to a 0° deflection – the quotient rises with decreasing deflection velocity, and takes substantially higher values following adaptation, suggesting that direction discrimination may improve with adaptation and may be more accurate for lower velocity deflections. However, biologically relevant direction classification must occur on a single-trial basis (as opposed to relying on a trial-averaged quantity); Figure 8 (middle) employs a linear classification scheme to assess the efficacy of a comparison between the 0° domain response/net cortical response quotient with the same quotient for the 45°/315° domain in correctly identifying deflection direction on a trial by trial basis (for a 0° deflection)—the post-adaptation classification rate is high and constant for all deflection velocities, while the pre-adaptation classification rate is lower, but rises with decreasing deflection velocity and approaches the (fixed) post-adaptation classification rate. This occurs as a consequence of the compound effect of adaptation and direction—both adaptation and deviations of deflection direction away from the preferred direction of an RS cell lead to decreases in the peak amplitude of TC input to the cell, drastically reducing the probability of an RS cell spike. Thus, following adaptation, the response of a direction domain drops drastically as its preferred direction deviates from the stimulus direction (with non-zero responses exhibited only by domains with preferred directions close to the stimulus direction (Figure 6, bottom right), and for non-optimal velocities, only the single direction domain whose preferred direction aligns with the stimulus direction exhibits a non-zero response); this results in a large difference in the domain response/net cortical response quotients for the 0° and 45°/315° domains and yields a high classification rate for all velocities. Prior to adaptation, high velocity deflections lead to substantial activity in all RS direction domains with considerable variability in individual RS cell responses (Figure 6, bottom left), leading to a relatively low classification rate, while as deflection velocity decreases, the responses of all RS direction domains diminish, but the non-linearity imposed by the spike threshold ensures that domains whose preferred directions deviate from 0° effectively display no spike response, leading to an increase in the classification rate (at the lowest velocity, only the 0° direction domain exhibits a significant non-zero response, yielding a high classification rate similar to the post-adaptation classification rate). This results in an overall substantial improvement following adaptation in the ability of the RS population response to pinpoint deflection direction (Figure 8, right).

## 4. Discussion

In this work, I construct a biologically-based model of the barrel cortex in which network parameters are sharply constrained by empirical measurements, and I examine individual RS cell and population responses to simulated whisker deflections of varying velocity and angular direction. Furthermore, I study the change in model dynamics after adaptation (which occurs biologically after low frequency repetitive whisker deflections) and the effects of adaptation on the accuracy of stimulus discrimination. Individual RS cell spiking probabilities are tuned for both deflection velocity and direction, with velocity (direction) tuning sharpening as deflection direction deviates from preferred (deflection velocity is lowered), and with sharper velocity/direction tuning in general following adaptation. Additionally, I investigate velocity and direction discrimination using a linear classifying scheme in a more biologically meaningful context (than single cell responses over multiple trials)—the ability of the cortical RS population to pinpoint deflection velocity or direction on a single-trial basis. Interestingly, this examination of single-trial stimulus discrimination via population responses shows that velocity discrimination changes little with adaptation, while direction discrimination improves substantially following adaptation.

While TC → FS, TC → RS, and FS → RS wiring and synaptic dynamics have been elucidated empirically (with experimental constraints incorporated in the model presented here), connectivity among RS cells and the dynamics of these synapses have not been well-characterized experimentally. RS → RS synapses, while included in the current model, do not affect the spiking behavior of RS cells, and hence RS → RS synaptic transmission is not analyzed in the results presented here. Rather, RS → RS synapses in the model serve to account for the experimentally observed direction-dependent timing and peak amplitude dynamics of excitatory and inhibitory inputs to RS cells (Wilent and Contreras, 2005), as detailed in prior work with this model (Patel, 2018). RS → RS synapses do not affect the spiking behavior of RS cells in the model due to the timing and amplitude dynamics of RS → RS input. Since RS input to an RS cell must await significant RS spiking within the barrel, RS → RS input is naturally delayed relative to TC input and tends to temporally coincide with potent FS input, and hence powerful simultaneous inhibition precludes the initiation of a spike (Patel, 2018). Furthermore, the strength of RS input to an RS cell depends on overall barrel activity (Patel, 2018); thus, following adaptation, while inhibition from FS cells to an RS cell weakens dramatically, the substantially reduced spiking activity of barrel RS cells ensures that RS → RS input is minimal and incapable of evoking an RS cell spike.

### 4.1. Role of adaptation

One possible functional interpretation of adaptation dynamics is that, prior to adaptation, high levels of RS cell spiking allow robust determination of whether or not a deflection has occurred, while after adaptation, increased sensitivity of RS cell responses allows for fine feature discrimination at the cost of robustness in the determination of the occurrence of a deflection (since, presumably, after repeated whisker deflections the animal is aware that a deflection has occurred and feature discrimination is the more relevant task) (Gabernet et al., 2005; Wang et al., 2010; Adibi et al., 2013a,b). The results of the present work suggest that discrimination (by population responses) of angular direction does indeed improve after adaptation, which may serve to enhance the acuity of barrel representations of object shape and texture post-adaptation. Another interesting possibility is that adaptation plays a role in the dynamics of whisker-whisker interactions. In natural roaming behavior, multiple whiskers are likely deflected repeatedly and concurrently, and hence in natural settings multiple barrels within somatosensory cortex are likely to be active simultaneously and in various states of adaptation. Adaptation, therefore, may serve to shape the functional interplay among different whisker barrels, sculpting activity patterns across somatosensory cortex and pinpointing novel deflection information that can guide moment-to-moment reallocations of attentional or processing resources. Moreover, the adapted state itself (as a means of signaling the occurrence of repeated whisker deflections) may provide information about environmental objects – different object properties (such as size, texture, and shape) can likely be inferred from a single deflection vs. repetitive deflections during natural roaming.

### 4.2. Model predictions

In concordance with prior experimental and theoretical observations (Wang et al., 2010; Adibi et al., 2013a,b; Liu et al., 2014), in the model presented here the velocity selectivity of the spike response of an individual RS cell sharpens following adaptation (Figure 3, right). However, the model also predicts that the velocity tuning of an individual RS cell sharpens as the angular direction of whisker deflection deviates from the preferred direction of the cell, a prediction that has not yet received experimental verification but can easily be tested via single-cell electrophysiological recordings. Moreover, the model predicts that the spike response of individual RS cells becomes more direction selective following adaptation (which can be tested through single-cell recordings as well), and the model accords with empirical data on direction tuning ratios showing that the direction selectivity of individual RS cells increases with decreasing deflection velocity (Bruno and Simons, 2002; Lee and Simons, 2004; Wilent and Contreras, 2005).

With the current model I also examine velocity and direction discrimination by population responses on a single-trial basis, a more biologically relevant context than trial-averaged responses of individual cells – since animals must perform stimulus discrimination based on a single trial, and individual RS cells tend to spike ~0–2 times per stimulus, the response of an individual RS cell is unlikely to be useful in single-trial stimulus identification. This context has not yet, to my knowledge, received experimental attention. The model predicts that, based on a simple linear classification scheme imposed upon single-trial population responses, velocity discrimination actually changes little with adaptation, but direction classification improves considerably following adaptation, a prediction that can be tested through multicellular recordings of cortical RS cells (and employment of a similar linear classifier on the resulting data).

There are several important caveats to the biological interpretation of the results presented here. The present model incorporates only 160 RS cells, while a biological barrel contains ~3600 RS cells (Bruno and Simons, 2002), and hence it is possible that the larger size of the rodent barrel could lead to biological population responses (if many or all barrel RS cells are measured) that differ from the population responses observed in the present model. Additionally, the rodent barrel system is responsive to multiple stimulus features, such as deflection amplitude and acceleration, in addition to the velocity and angular direction of a deflection of the primary whisker. Furthermore, RS cells within a barrel can display complex and varied feature selectivity, with many RS cells integrating input from several whiskers (Bale and Maravall, 2018). While the model studied in this paper incorporates realistic and randomly distributed TC → RS cell connectivity, giving rising to significant variability in the responses of RS cells even within a particular direction domain, it incorporates only stimulus velocity and angular direction of primary whisker deflection and does not include the full variability in feature selectivity properties and whisker receptive fields across RS cells seen experimentally. Since the model in this work is specifically employed to assess single-trial stimulus discrimination using RS population responses, the reduced variability among RS cells in the model implies that the results of the present work must be translated with caution when examining population responses in a biological barrel. Moreover, the dynamics of RS → RS synapses have not been well-characterized experimentally, and while in the model, as described in Patel (2018), they play a pivotal role in explaining the direction dependence of excitatory and inhibitory input to an RS cell (Wilent and Contreras, 2005) without affecting RS cell spiking behavior, in a biological barrel they may also play an as-of-yet undeciphered role in sculpting long-term barrel dynamics or affecting variability in response properties among RS cells. However, there are likely numerous RS cells within a biological barrel that exhibit selectivity to velocity and angular direction of primary whisker deflection similar to those in the model presented in this paper, and aggregate responses from such cells can be employed to test the predictions of the model (additionally, it reasonable to expect that such cells play the most prominent role in the biological discrimination of these stimulus features).

### 4.3. Other models

Prior modeling work carried out within the barrel system has been aimed at elucidating response properties of barrel neurons in the context of thalamic and cortical input, delineating the interplay of synaptic and intrinsic neuronal properties, describing the transformation of receptive fields from barreloid to barrel cells, and the computation of temporal intervals between the deflections of distinct whiskers (Kyriazi and Simons, 1993; Pinto et al., 1996, 2003; Pesavento et al., 2010; Wilson et al., 2011; Ly et al., 2012; Middleton et al., 2012; Pesavento and Pinto, 2012; Liu et al., 2014). The modeling work in this manuscript follows a similar modeling philosophy to that of Kyriazi and Simons (1993) and Liu et al. (2014), in that I do not attempt to incorporate the detailed intrinsic neuronal properties of TC, FS, and RS cells; rather, I construct a biologically-oriented model that captures network structure and dynamics, with model parameters sharply constrained by physiological measurements. In contrast to prior models, the present study simulates both the velocity and angular direction of whisker deflection, studies the responses of RS cells with varying direction preference to both of these critical stimulus features, and examines stimulus classification by population activity on a single-trial basis.

Experiments show that as the angular direction of whisker deflection deviates from the preferred direction of an RS cell, the peak amplitude of excitation to the RS cell diminishes only minimally while the timing of the peak in excitation shifts toward the delayed (and fixed over deflections directions) peak in incoming inhibition (Wilent and Contreras, 2005). In accordance with these results, an earlier modeling study employs a single integrate-and-fire RS cell and simulates whisker deflections of varying angular direction by altering the amplitude and timing of excitatory input (relative to inhibitory input) to the RS cell, and the authors show that repetitive whisker deflection at high frequency (~200 Hz) degrades direction tuning due to the time scale of the interdeflection interval being smaller than time scale over which the RS cell integrates excitatory input (Puccini et al., 2006). This is not inconsistent with the results of the present work, since in the current work I simulate adaptation by altering synaptic strengths within the network in accordance with adaptation dynamics observed with repetitive whisker deflections at low frequency (~20 Hz) (Gabernet et al., 2005; Temereanca et al., 2008)—at such low repetition frequencies, interdeflection intervals are larger than the integration time scale of an RS cell, and hence a loss of RS cell direction tuning due to summation of inputs from successive deflections would not be expected. Indeed, in Puccini et al. (2006) the authors show that, in their model, simulation of low frequency repetitive input does not lead to a degradation in direction tuning of the RS cell.

While the model of Puccini et al. (2006) suggests that the direction tuning of a single RS cell changes little after low frequency repetitive stimulation, the model in the present study suggests that RS cell direction tuning markedly sharpens. However, evidence indicates that, following adaptation with low frequency repetitive deflections, the probability of an RS cell spike diminishes substantially (Gabernet et al., 2005; Temereanca et al., 2008) and velocity tuning sharpens (Wang et al., 2010; Adibi et al., 2013a,b; Liu et al., 2014); this suggests a plausible scenario in which the non-linear effect of the spike threshold causes a more dramatic decline in the response to non-preferred vs. preferred directions following low frequency adaptation (in the same manner that a threshold effect preferentially diminishes the response to low velocity stimuli following low frequency adaptation). Furthermore, the model of Puccini et al. (2006) *phenomenologically* constructs excitatory and inhibitory inputs to an RS cell in order to incorporate the direction dependence of input timing and amplitude observed experimentally (Wilent and Contreras, 2005), without suggesting a mechanism through which this direction dependence arises; the present model, on the other hand, incorporates a simple and biologically plausible mechanism based on RS → RS synapses to explain these empirical observations, as described in detail in prior work with this model (Patel, 2018).

### 4.4. Future work

The encoding of both stimulus velocity and angular direction by the spike rate (or probability) of RS cells, both biologically and in the model presented here, leads to a natural conundrum—how does the barrel cortex disentangle RS cell responses and pinpoint the identity of these two stimulus features if they are both encoded by the same variable? The answer likely lies in the fact that all RS cells exhibit the same velocity dependence, while direction tuning varies among RS cells—lowering deflection velocity lowers the spike response of all barrel RS cells, while changing deflection direction elevates the response of some RS cells while diminishing the response of others (depending on RS cell direction preference). This suggests that stimulus velocity and direction classification is likely based on population responses, and future work will investigate, in a biophysical model, the manner by which population responses may be used by the barrel cortex to disentangle these two stimulus features and separately identify both stimulus velocity and angular direction.

## Author contributions

The author confirms being the sole contributor of this work and approved it for publication.

### Conflict of interest statement

The author declares that the research was conducted in the absence of any commercial or financial relationships that could be construed as a potential conflict of interest.
